# Bead-Immobilized Multimodal Molecular Beacon-Equipped DNA Machinery for Specific RNA Target Detection: A Prototypical Molecular Nanobiosensor

**DOI:** 10.3390/nano11061617

**Published:** 2021-06-20

**Authors:** Jeonghun Kim, So Yeon Ahn, Soong Ho Um

**Affiliations:** 1 School of Chemical Engineering, Sungkyunkwan University, Suwon 16419, Gyeonggi-do, Korea; realread2@naver.com (J.K.); melissa100@naver.com (S.Y.A.); 2 SKKU Advanced Institute of Nanotechnology (SAINT), Sungkyunkwan University, Suwon 16419, Gyeonggi-do, Korea

**Keywords:** molecular beacon, DNA nanostructure, miRNA, sensor

## Abstract

A variety of nanostructured diagnostic tools have been developed for the precise detection of known genetic variants. Molecular beacon systems are very promising tools due to their specific selectivity coupled with relatively lower cost and time requirements than existing molecular detection tools such as next generation sequencing or real-time PCR (polymerase chain reaction). However, they are prone to errors induced by secondary structure responses to environmental fluctuations, such as temperature and pH. Herein, we report a temperature-insensitive, bead-immobilized, molecular beacon-equipped novel DNA nanostructure for detection of cancer miRNA variants with the consideration of thermodynamics. This system consists of three parts: a molecular beacon for cancer-specific RNA capture, a stem body as a core template, and a single bead for solid-support. This DNA system was selectively bound to nanosized beads using avidin–biotin chemistry. Synthetic DNA nanostructures, designed based on the principle of fluorescence-resonance enhanced transfer, were effectively applied for in vitro cancer-specific RNA detection. Several parameters were optimized for higher performance, with a focus on thermodynamic stability. Theoretical issues regarding the secondary structure of a single molecular beacon and its combinatory forms were also studied. This study provides design guidelines for new sensing systems of miRNA variation for next-generation biotechnological applications.

## 1. Introduction

Genomics has guided the identification of numerous genetic variants associated with cancers [1,2]. This has spurred the development of genetic analytic tool kits with high sensitivity and selectivity for detecting such variants. A number of applications have recently been developed in relation to these genetic analytical tools. Some of them show breakthrough detection ability. Most of these applications are focused on increasing the sensitivity itself, such as amplifying the target or amplifying the signal [3,4,5,6,7]. In particular, molecular beacons, consisting of DNA hairpins with a fluorescent label at one end and quenchers at the other end, are widely used because they obviate the requirement to label the target molecule. Molecular beacon technology is a groundbreaking technology that easily detect target biomarkers by simple mechanism. However, hairpin structure without rigid support is less stable and it makes high background signal. Therefore, it is commonly used that immobilize hairpin structure onto solid substrates [8]. Although surface-immobilized molecular beacons have been mainly reported [9], most studies have simply described a one-to-one correspondence to the read-out of targets. The critical challenge faced by immobilized molecular beacons is precisely controlling the density and orientation of grafted DNA probes to minimize the interactions between probes and to maximize the target accessibility [10]. Herein, we demonstrate a new surface-immobilized molecular beacon sensor employing a robust DNA nanostructure that enables precise signal adjustment and probe representation. The DNA nanostructure-integrated molecular beacon system is able to capture a target miRNA marker and simultaneously provide quantitative indication of probe internalization that can function as a part of a target-label-free optical genetic biosensor. The optimized DNA nanostructure contributed to development of an advanced molecular beacon sensor enabling quantitative detection of breast-cancer specific RNA markers.

## 2. Materials and Methods

All nucleic acids, including DNAs (Y_1,_ biotin-labeled Y_1_, FAM-labeled Y_2_, Y_3_, Y_L_), mismatched DNAs, and RNAs (EZH2, miRNA 21) were purchased from Integrated DNA Technologies (Coralville, IA, USA). Y_L_ modified with both dark quencher (Iowa black RQ) at the 5′ end and cyanine dyes (Cy5) at an intra-molecular location was purchased from Integrated DNA Technologies. Agar powder was purchased from Sigma-Aldrich (St. Louis, MO, USA). Chemicals used in this experiment, which included ethidium bromide, a 25 bp DNA stepladder, 50 bp DNA ladder, and 6× blue/orange loading dye, were purchased from Promega (Fitchburg, WI, USA). TAE buffer (50×) and TBE buffer (10×) were purchased from Noble Biosciences (Suwon, Gyeonggi-do, Korea). SYBR gold nucleic acid gel stain (10,000×) and ultrapure Dnase/Rnase-free water were purchased from Thermo Fisher Scientific (Waltham, MA, USA). Streptavidin-coated polystyrene beads (110 nm-sized) were purchased from Bangs Laboratories (Fishers, IN, USA).

### 2.1. Design and Evaluation of DNA Sequences Using Web-Based Analysis Programs

The thermodynamic energies of all oligonucleotides used in this experiment were investigated via OligoAnalyzer 3.1 provided by Integrated DNA Technologies. In addition, thermodynamic energies of hairpin-looped structures of oligo candidates were investigated using Beacon Designer web-based software provided by Premier Biosoft International. Sequences and other factors, such as temperatures and salt concentrations, were entered into the software. Here, we designed several sequences as follows. Each sequence is also described in Figure S3.

Y-DNA, y shaped DNA structure which composed by Y_1_, Y_2_, Y_3_
L-DNA, y shaped DNA structure with loop region which composed by Y_1_, Y_2_, Y_L(loop)_

### 2.2. Synthesis of L-DNA

Lyophilized oligonucleotides were separately redissolved in TE buffer (10 mM Tris-HCl (pH 8.0) and 0.1 mM EDTA). The optical density (OD) of each oligo solution was measured by a UV-Vis photometer (BioPhotometer, Eppendorf, Hamburg, Germany), which indicated the concentration in μg/μL. Each solution was stored at 4 °C. To prepare 6 μM Y- and L-DNA solutions, each oligonucleotide was mixed in a buffer composed of 50 mM NaCl, 10 mM Tris-HCl (pH 8.0), and 0.1 mM EDTA. The mixture was put into a thermal cycler (Mastercycler Pro, Eppendorf) and annealed with temperature- and time-varying processes. Samples were then heated to 95 °C for 2 h, followed by cooling to 65 °C for 5 min and further cooling to 60 °C for 5 min. The temperature was then reduced by 1 °C per minute until it reached 20 °C. Samples were stored at 4 °C until use.

### 2.3. Prediction of Possible Secondary Structures of L-DNA through Web-Based Software

Thermodynamic properties and secondary structures of each oligonucleotide were obtained using the free Mfold program. The gateway for the Mfold web server is http://unafold.rna.albany.edu/, accessed on 2 September 2008. Predicted secondary structures in one- and two-dimensions were obtained under the following conditions: 6 μM oligos, 50 mM NaCl, and 37 °C or 50 °C. 1-D data were further used to construct a three-dimensional model using RNA composer (http://rnacomposer.cs.put.poznan.pl/, accessed on 26 April 2012). The RNA composer output was saved in pdb format, and collected images were produced using PyMOL software (http://www.pymol.org, accessed on 1 August 2006).

### 2.4. Melting Curves of Oligonucleotide Sequences

The thermal denaturation of oligos was measured by the 7500 Real Time PCR System manufactured by Applied Biosystems (Foster City, CA, USA) and analyzed using 7500 software V2.3. SYBR Green I from Molecular Probes (Eugene, OR, USA) was used as a nucleic acid staining agent. SYBR Green I solution, which was diluted 20,000-fold, was added to 600 ng of DNA solution in a final volume of 100 μL on a 96-well PCR plate. Melting curves were collected in the SYBR channel using the ramp rate of +0.5 °C/min from 30–95 °C. Fluorescence measurements were performed at each step during this ramp. Melting curves were performed at least in triplicate.

### 2.5. Gel Electrophoresis and Single-Stranded Conformational Polymorphism (SSCP) Analysis

DNA products were evaluated by 3% agarose gel electrophoresis. After DNA samples were synthesized, 0.6 μg of each sample was added to 12 μL of loading buffer (2 μL of 6× loading dye with DNase-free water). Gel electrophoresis was performed at 100 V for 40 min, and gels were then immediately stained with ethidium bromide (EtBr) (2 μg/mL) for 20 min. For single-stranded conformational polymorphism (SSCP) analysis, polyacrylamide gel electrophoresis was performed. Products (0.3 μg of DNA sample) were evaluated by 15% polyacrylamide gel electrophoresis. Gel electrophoresis was performed at 100 V for 1 or 2 h, and then gels were immediately stained with SYBR gold for 30 min. The fluorescent intensity of FAM under UV light was evaluated for 0.3 μg L-DNA and other samples. Gel images were visualized using the GELDoc-it imaging system combined with a Launch VisionWorksLS UPV and were analyzed using a TotalLab Quant gel quantification software version 2.01, provided by ImageMaster of TotalLab Ltd. (Newcastle, UK).

### 2.6. Measurement of Thermal Stability and Detection Efficiency of L-DNA and Its Derivatives

Thermal stability at 9, 16, 25, 37, and 45 °C was measured using a SpectraMax M5 (Molecular Devices, Sunnyvale, CA, USA). Thermal stability of 1.2 μM of L-DNA and its derivatives was measured in a 40 μL final volume on a 384-well plate. To investigate detection efficiency, 5 μL of 1.2, 0.6, and 0.12 μM complementary target mRNA EZH2 was added to 40 μL of L-DNA and its derivatives. After incubation for 30 min at 37 °C to allow binding to target molecules, fluorescence of the mixtures was measured using a SpectraMax M5. The Cy5 signal was measured at wavelengths of 648 nm and 675 nm. Fluorescence measurements were obtained from triplicate experiments and sample standard deviation was calculated for statistical evaluation.

### 2.7. Preparation of a Bead-Immobilized Molecular Beacon-Equipped DNA Nanobiosensor

Free molecular-beacon-equipped DNA nanosensors, which we also refer to as L-DNA, was added to a solution of 110 nm PS beads to construct bead-immobilized molecular beacon-equipped DNA nanobiosensors. This PS bead solution was provided by Bangs Laboratories (Fishers, IN, USA) and diluted in PBS (0.05% Tween 20) at a 1% volumetric ratio. 50 μL of the 6 μM L-DNA solution was added to 500 μL diluted bead solution at a 10:1 volumetric ratio. The mixture was then incubated for 1 h at room temperature. After sequential reactions, the samples were centrifuged at 10,000 g for 30 min at 4 °C, followed by two washes with 500 μL of PBS and resuspension in 50 μL of buffer (50 mM NaCl, 10 mM Tris-HCl (pH 8.0), and 0.1 mM EDTA). 50 μL of L-DNA beads were prepared. To investigate detection efficiency, 2 μM of L-DNA and bead-immobilized L-DNA with 2, 0.5, 0.125, 0.03125, and 0 μM of the miRNA-21 were prepared in 40 μL of 50 mM NaCl, 10 mM Tris-HCl (pH 8.0), and 0.1 mM EDTA in a 384-well plate. Fluorescence of the mixtures was measured by a SpectraMax M5 at 20 °C. FAM and Cy5 were measured at excitation wavelengths of 495 and 648 nm and emission wavelengths of 525 and 675 nm, respectively. Using FAM as a standard, detection efficiency was evaluated as the relative Cy5/FAM signal variation. The binding capacity of 110 nm-sized streptavidin-coated microspheres was estimated to be approximately 3.6 μg biotin-FITC/mg microspheres. Considering that the molecular weight of the biotin-FITC is around 831 Da, the number of microspheres per gram was 1.391 × 10^15^. This value indicates that one microsphere has approximately 1875 biotin-FITC particles attached to it.

### 2.8. pH and Temperature Stability Measurements of Bead-Immobilized Molecular Beacon-Equipped DNA Nanobiosensors

The stability of bead-immobilized molecular beacon-equipped DNA nanobiosensors was tested in different pH environments. When a loop–stem structure formed, Cy5 reacted with the Iowa Black RQ quencher, silencing fluorescence of the cyanine dye. After fabrication of the L-DNA-bead system, it was exposed to solutions with different pH values: pH 5.5 (50 mM Tris-acetate), 6.5 (50 mM MES), and 7.4 (50 mM Tris-HCl) in a 100-μL final volume in a 96-well PCR plate. Melting curves were collected in the Cy5 channel using a ramp rate of +0.02 °C/sec from 30 °C to 95 °C. Fluorescence measurements were performed at each step during the ramping process. Thermal and environment stability of the system was measured by a LightCycler 480 II Real-Time System provided by Roche Diagnostics and was analyzed using the LightCycler Adapt Software v1.1 from Roche Diagnostics. Cy5 fluorescence was measured with an excitation wavelength of 650 nm and an emission wavelength of 670 nm.

### 2.9. Melting Curve Measurements of L-DNA Sequences and Their Derivatives

Thermal denaturation was measured by the 7500 Real Time PCR System from Applied Biosystems (Foster City, CA, USA) and analyzed using 7500 software V2.3. SYBR Green I from Molecular Probes (Eugene, OR, USA). SYBR Green I solution was diluted 20,000× and added to 600 ng of DNA solution in a 100 μL final volume in a 96-well PCR plate. Melting curves were measured in the SYBR channel using a ramp rate of +0.5 °C/min from 30 to 95 °C. Fluorescence measurements were performed at each step during this ramping process. Melting curves were performed at least in triplicate.

### 2.10. Evaluation of the Mole Fraction of Complementary Hybridization via UNAFold Software

All single-stranded DNA sequences were saved in fasta file format. In a terminal command window, two single-stranded DNA files were opened. The molar concentrations of each sequence and the reaction salt concentrations were designated. In our analysis, 6 μM DNA was tested. Yield from DNA hybridization was analyzed at sodium ion concentrations ranging from 50 mM to 500 mM. After running the program, mole fractions of each component according to reaction temperature were obtained. Further details can be found in the protocol manual on the following website: http://unafold.rna.albany.edu.

### 2.11. Trend Line Drawing via CurveExpert Basic Software

The graphs according to fluorescence data in figures (i.e., in Figure 5b,c)were drawn by the CurveExpert program, which is based on the Weibull model among the sigmoidal functions. The equation of the Weibull model is written as below.

(1) $$y=a-b\times exp\left(-cx^{d}\right)$$

The a is an integer, b is $\frac{d}{\lambda }{\left(\frac{x}{\lambda }\right)}^{d-1}$, c is ${\left(-\frac{1.}{\lambda }\right)}^{d-1}$, d expressed the order of events, and λ is a number of frequencies. For detailed description and use of the program, please refer to the website: http://www.curveexpert.net.

## 3. Results and Discussion

**Figure 1:**
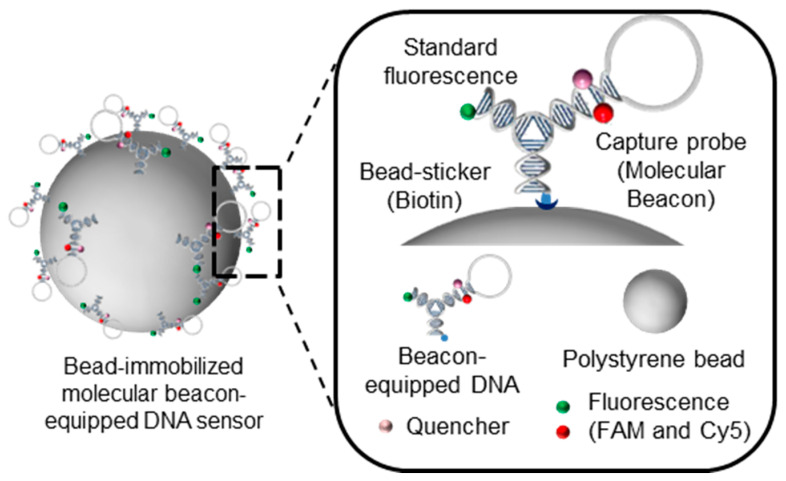
Schematic of the multimodular bead-immobilized molecular beacon-equipped DNA nanosensor. A looped DNA fragment that captures target RNA molecules was added onto a one-component single-stranded DNA molecule.

To achieve multi-functionality in the molecular beacon system, a tree DNA-based nanostructure was used as a template [11,12]. Y-shaped DNA (Y-DNA) with three arms, each containing a different module, allows the body to have three different properties [13,14]. Multimodularity can be attained via a one-pot multistep organic synthesis [15]. As a test model for this study, we modified each arm of Y-DNA to have distinct functional modules and named the nanostructure looped DNA nanostructure (L-DNA). The modules on each arm are molecular beacons, consisting of a hairpin structure with Cy5 fluorophore and a dark quencher, biotin as a bead-sticker, and fluorescein phosphoarmidite (FAM) as a standard fluorescence (Figure 1). These functional modules in each arm allow for simultaneous detection of target molecules and fine-tuning of signal-off for target quantification. The original Y-DNA is composed of Y_1_, Y_2_, and Y_3_ strands, and biotin, FAM, and molecular beacon are attached to the 5′-end of each strand respectively on L-DNA. The molecular beacon attached Y_3_ strand is named as Y_L_. The stability of L-shaped DNA can be affected by unexpected structural patterns caused by variations in the stem-loop internal structure. We therefore used the web-based software OligoAnalyzer 3.1 and Beacon Designer to design L-shaped DNA (Figure S1 of the online supplementary material). We focused on GC content, melting temperature (T_m_), and secondary structure to minimize the possibility of formation of secondary structures due to non-specific binding interactions between the L-DNA and the target sample.

**Figure 2:**
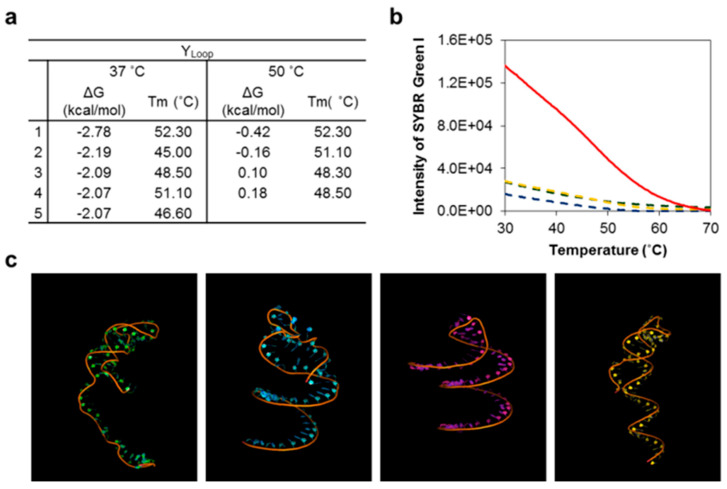
Empirical and theoretical analyses of possible secondary structures of L-DNA. (**a**) Theoretical analysis of the secondary structures of L-DNA via the Mfold program. Y_L_, which contains a loop–stem form, exhibited possible secondary structures with high thermodynamic stability at both 37 °C and 50 °C. Both Gibbs free energy and T_m_ values of its secondary structures are described in the table. (**b**) Melting curves of Y_1_ (green), Y_2_ (blue), Y_3_ (orange), and Y_L_ (red) as measured by RT-PCR. The graph indicates changes in fluorescence intensity of SYBR green I, which is specific to double-stranded DNAs. Data were obtained from triplicate experiments. (**c**) Three-dimensional topographic images of four different types of possible secondary structures of Y_L_ at 50 °C.

The efficiency of L-DNA can be altered by the formation of unexpected secondary structures. Therefore, it is important to optimize its structure in consideration of several environmental factors that affect thermodynamic stability (Figure S2). In particular, a more homogeneous complex can be obtained if possible secondary structures at different temperatures are evaluated. We investigated the thermal stability of L-DNA via both experimental and theoretical analyses of individual sequences (Figure 2 and Figure S3). Y-shaped DNA functionalized with one molecular beacon was assessed theoretically using the mfold program. The T_m_ values of each oligonucleotide were measured by real-time PCR. In the temperature range from 37 to 50 °C, both Y_1_ and Y_2_ were predicted to form non-secondary structures because of their length of 26 bases, whereas Y_L_, which was 57 bases long and complementary to the target RNA sample, formed several different secondary structures.

**Figure 3:**
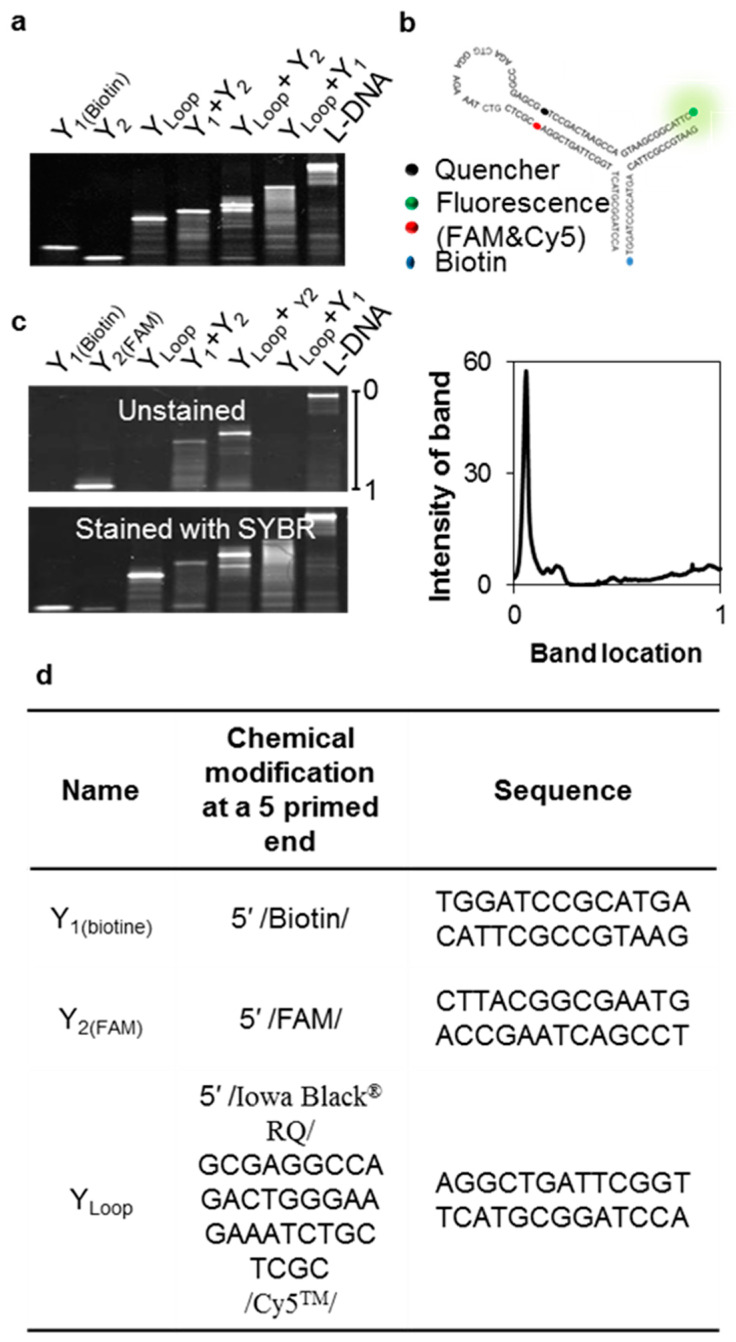
Evaluation of state-runs of L-DNA by single-strand conformation polymorphism (SSCP) analysis. (**a**) SSCP analysis of L-DNA and its partial constructs. Sequential build-ups of L-DNA were subjected to 15% polyacrylamide gel electrophoresis at 100 V for 2 h, followed immediately by specific staining with 2 μg/mL SYBR gold for 30 min. (**b**) Structural form of as-predicted L-DNA. (**c**) Gel electrophoretic image of a set of FAM-labeled L-DNAs. FAM-labeled single-stranded DNA fragments were present in all L-DNAs. After fluorescence intensity measurements, the yield of L-DNA was calculated using TotalLab Quant gel quantification software. Minimum profile was then used as a single background; the total yield of synthesized L-DNA was 62.6%. (**d**) Sequence information for the fragments used in this experiment.

**Figure 4:**
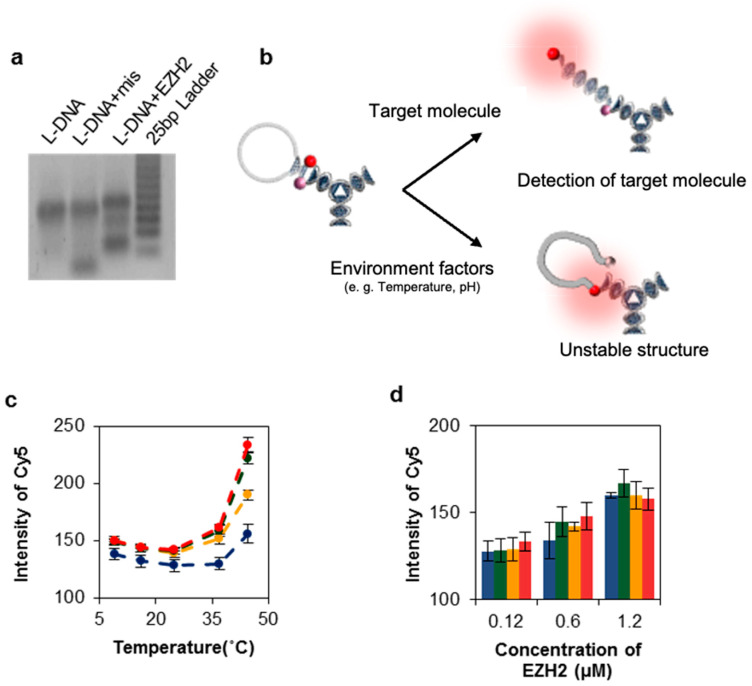
Evaluation of both thermodynamic stability and detection efficiency of L-DNA and its partial constructs. (**a**) Specific interaction between L-DNA and its complementary pair in a 3% agarose gel. The same amounts (6 μM) of mismatched oligonucleotide (mis, 5′- CAT ATG GGC TCC ATC GGC GCA-3′) and fully matched oligonucleotide (5′-CAG ATT TCT TCC CAG TCT GGC-3′) were used for comparison. (**b**) Schematic of the detection mechanism of L-DNA in solution. L-DNAs formed a looped structure in molecular beacon target capture. After target binding, the beacon adopted an open state, emitting a strong Cy5 signal. Environmental factors such as temperature and pH can destabilize the loop–stem structure and can affect the fluorescence signal. (**c**) Cy5 signal of L-DNA (red), Y_L_ + Y_1_ (orange), Y_L_ + Y_2_ (green), and Y_L_ (blue) at different temperatures (9, 16, 25, 37, and 45 °C). (**d**) Cy5 signal variations of L-DNA at different target concentrations at 16 °C. In both (c) and (d), the fluorescent intensities of Cy5 measured by spectrophotometric analysis were obtained from triplicate experiments.

This outcome was also confirmed experimentally. Interestingly, Y_L_ alone showed stronger emission signals (~4-fold) than the other Y_n_ at around 60 °C. No fluorescent emissions induced by detection were found from single components of the Y_n_ samples. We studied this further using build-ups of single-stranded DNA fragments, including the complexes of Y_1_ + Y_2_, Y_1_ + Y_L_, Y_2_ + Y_L,_ and Y_1_ + Y_2_ + Y_L_ (Figure S4). Most DNA constructs were synthesized below 60 °C (T_m_ = 56.5 °C). Production efficiency was investigated by varying temperatures around T_m_ (Figure S4b,c). Ideally, the binding fractions of oligo-fragments in each complex should be equal, and the total yield fraction should be 1.0. Y_L_ + Y_1_ and Y_L_ + Y_2_ had molar yield fractions of 0.76 and 0.53, respectively. The total proportion of unreacted fragments was 23.5% because the secondary structure of the longer Y_L_ fragment could inhibit the hybridization of complexes. This result was confirmed by single-stranded conformational polymorphism (SSCP) analysis through a gel-electrophoretic migration shift assay (GEMSA) using a 15% polyacrylamide gel (Figure 3) [16]. Staining of the gel with SYBR gold revealed that Y_L_ had adopted a secondary structure conformation, and that the complete set of L-DNA complexes was fragmented [17,18]. Both Y_1_ and Y_2_ appeared as single bands (Figure 3a), but Y_L_ was characterized by several bands, indicating potential secondary structures. After annealing, a set of DNA complexes involving Y_1_, Y_2_, Y_L_, Y_1_ + Y_2_, Y_1_ + Y_L_, and Y_2_ + Y_L_ were represented by several bands, indicating the secondary structures of these complexes. Y_L_ was specifically labeled with fluorescein (FAM), and roughly 62.6% of Y_L_-containing product was obtained (Figure 3b,c). This result confirms that Y_L_ plays a significant role in product synthesis by competing with the formation of either secondary structures or products. To effectively recognize and capture a complementary RNA target, the loop structure of L-DNA was further examined (Figure 4). An open-close state of the loop on Y_3_ of L-DNA can be used for specific detection of an RNA target via fluorescence-resonance enhanced transfer (FRET), in which FRET signals are emitted as complementary RNA pairs with looped capture sequences (Figure 4). Working on-signaling may be strongly affected by environmental conditions such as target mass and reaction temperature. After L-DNA was synthesized, the T_m_ of the loop–stem structure was different from the as-expected T_m_ of 48 °C (Figure 4c); the T_m_ was around 37 °C, as determined by tracing the increase in the fluorescence of Cy5 as the fluorophore. In the case of the Y_L_ sequence, the Cy5 signal increased at around 45 °C. In contrast, L-DNA and its byproducts such as Y_L_ + Y_1_ and Y_L_ + Y_2_ exhibited an increased Cy5 signal at 37 °C, indicating that the loop–stem compartment was partially opened. This result implies that the loop–stem part of the L-DNA had a different thermal stability than Y_L_ alone. It is speculated that the detection resolution of L-DNA could be interrupted. It is strongly recommended not to use the L-DNA at temperatures below 30 °C. We also investigated that the yield of L-DNA formation increased at high salt concentrations (Figure S5).

Prior to in vitro detection verification of the functionality of the synthesized L-DNAs, we examined the detection efficiency of L-DNA and its partial derivatives in solution. Signal increments of Cy5 for L-DNA and its derivatives (Y_L_ + Y_1_, Y_L_ + Y_2_ and Y_L_) were tested by varying the masses of EZH2 mRNA, which is a breast cancer-specific biomarker (Figure 4d) [19,20]. Following the optimized procedure, 5 μL of EZH2 mRNA at concentrations of 0.12, 0.6, and 1.2 μM were slowly mixed into 40 μL of sample DNA nanostructures at 1.2 μM, after which the intensity of Cy5 was measured at 16 °C. In Figure 4c, as an example of disassembling the loop structure to characterize the on-signaling reaction, the loop structures in various combinations are thermally agitated. The alteration made in Cy5 intensity is much larger in case of L-DNA when compared to other combinations, which brings efficient on-signaling result from loop disassembly. As continued in Figure 4d, the favorable loop disassembly of L-DNA was then induced by the target EZH2 RNA, which resulted in better distinction between the signal from 0.12 µM and 0.6 µM.

**Figure 5:**
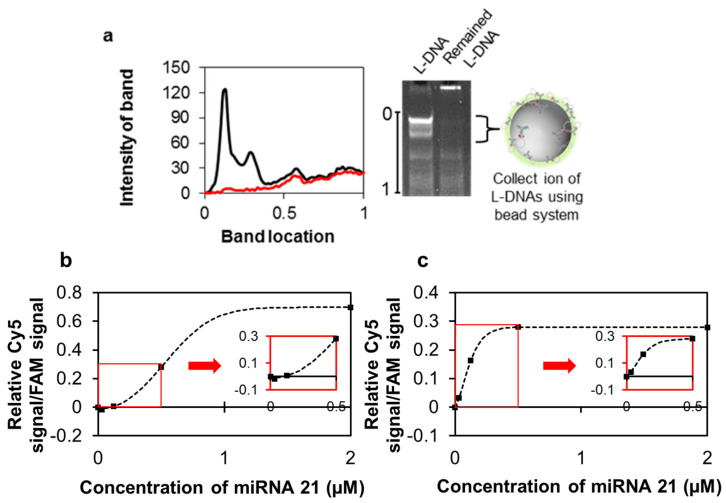
Construction of a bead-immobilized molecular beacon-equipped DNA nanosensor and its detection efficiency. (**a**) 15% PAGE image of the molecular-beacon-equipped DNA nanosensor (L-DNA) and the rest of L-DNAs. L-DNA was collected by 110 nm-sized PS beads via avidin–biotin conjugate chemistry. Red and black lines indicate L-DNAs and remaining L-DNAs, respectively. Minimum profile was used as a background signal; the purity of L-DNA increased from 58.4 to 86.8% when a solid support was used. (**b**) Detection efficiencies of free L-DNA and (**c**) the bead-immobilized L-DNA nanosensor system were compared. All data collected were obtained from triplicate experiments. The miRNA 21 was used at concentrations of 2, 0.5, 0.125, 0.03125, and used 0 μM as a negative control.

L-DNA was then applied for detection of cancer-specific microRNAs as a proof of concept for a potential cancer diagnosis platform, with internal fluorescent signals transmitted in situ. To test a miRNA model, miRNA21 was selected, and its complementary DNA sequence was incorporated into the L-DNA. Biotin-modified L-DNA was then conjugated onto streptavidin-coated polystyrene beads (PS beads) with a diameter of 110 nm via the avidin-biotin interaction (Figure 5 and Figure S7). Optimal parameters for the bead-immobilized L-DNAs were determined by varying conditions such as temperature and pH (Figure S8). The detection efficiencies of free L-DNA and bead-immobilized L-DNA were compared using FAM-tagged L-DNAs (sample preparation method described in the Supplementary Information). Fluorescence was monitored at different concentrations (2, 0.5, 0.125, 0.03125, and 0 μM) of miRNAs (Figure 5b,c). FAM was used as a standard, and the relative Cy5 signal/FAM fluorescence signal was obtained. The bead-immobilized L-DNA system could detect very low quantities of miRNA21 (as low as 0.03125 μM). Bead immobilization of L-DNA was possible up to 2000 units of L-DNA, leading to amplified fluorescence signals. The bead-immobilized system improved L-DNA detection efficacy in comparison to free L-DNA with regard to both sensitivity and specificity. However, as shown in Figure 5a, a large amount of L-DNA was lost during bead and L-DNA conjugation, so the total amount of L-DNA between the two control groups was different. Therefore, the fluorescence value did not increase after 0.5 μM concentration, even if more miRNAs were given.

## 4. Conclusions

In conclusion, we described a DNA nanostructure-based miRNA detector under consideration of thermodynamical stability of the nanostructure and probe for maximized interaction between probe and target, signal adjustment, and immobilization. Thermodynamic stability of DNA nanostructure scaffolded probe was analyzed and optimized empirically and theoretically. Diagnostic utility of an optimized DNA nanostructure was explored for detection of small quantities of a breast-cancer-specific RNA marker; our detection system was 16-fold more sensitive than conventional systems. We are currently adapting this diagnostic platform for detection of other genetic variants associated with diseases.

## Data Availability

Not applicable.

## Supplementary Materials

The following are available online at https://www.mdpi.com/article/10.3390/nano11061617/s1, Figure S1: theoretical evaluation regarding the design of loop sequence (Y_L_), a single-stranded oligo-fragment on a molecular beacon-equipped DNA nanobiosensor, Figure S2: algorithm diagram for optimized parameters for the synthesis of a bead-free nanobiosensor, Figure S3: sequence information for template DNA and bead-free nanobiosensor or L-DNA, Figure S4: melting curve analysis of DNA derivatives, Figure S5: yield increment in L-DNA according to salt concentrations, Figure S6: detection efficiencies of L-DNA and its derivatives, Figure S7: synthesis of a bead-immobilized molecular beacon-equipped DNA nanobiosensor system and confirmation of unreacted sequences, Figure S8: environmental stability of bead-immobilized nanobiosensor system, and Supplementary references.

## Table of Contents Graphic

The bead-immobilized multimodular molecular beacon-equipped DNA nanobiosensor is established; Immobilization of this detector amplified fluorescence signals substantially with selectivity when specific target RNA was captured, and these signals are simultaneously normalized by standard fluorescence.
