# Supplementary material for: Bead-Immobilized Multimodal Molecular Beacon-Equipped DNA Machinery for Specific RNA Target Detection: A Prototypical Molecular Nanobiosensor
Source: Nanomaterials (Basel). 2021 Jun 20;11(6):1617. doi: 10.3390/nano11061617 (PMC8235652; doi:10.3390/nano11061617)
Supplement: Supplementary file 1 [file nanomaterials-11-01617-s001.zip › nanomaterials-1222453-supplementary.pdf]

nanomaterials

Supplementary Materials

Bead-Immobilized Multimodal Molecular Beacon-Equipped DNA Machinery for Specific RNA Target Detection: A Prototypical Molecular Nanobiosensor

Jeonghun Kim [1,†], So Yeon Ahn [1,†] and Soong Ho Um [1,2,*]

[1] School of Chemical Engineering, Sungkyunkwan University, Suwon 16419, Gyeonggi-do, Korea; realread2@naver.com (J.K.); melissa100@naver.com (S.Y.A.)
[2] SKKU Advanced Institute of Nanotechnology (SAINT), Sungkyunkwan University, Suwon 16419, Gyeonggi-do, Korea
* Correspondence: sh.um@skku.edu
† These authors equally contributed to this work.

a

	**Stem part**	**70~80% of GC contents**		**GC contents of primer (%)**
		**G or C pair**	**A or T pair**	
	4 Base pairs	3	1	58.6
	5 Base pairs	4	1	61.3
	6 Base pairs	4	2	57.6
		5	1	-
	7 Base pairs	5	2	60
		6	1	-

b

**4 Base pairs**				**5 Base pairs**					
**Stem**	**T_m**	**Stem**	**T_m**	**Stem**	**T_m**	**Stem**	**T_m**	Stem	T_m
GGAG	N.M	GCAG	↓ 37 °C	GGCAG	48 °C	GCGAG	U.W.	GCCAG	47.1 °C
GGAC	N.M	GCAC	N.M	GGCAC	U.W.	GCGAC	U.W.	GCCAC	U.W.
GGTG	N.M	GCTG	↓ 37 °C	GGCTG	50.2 °C	GCGTG	47.1 °C	GCCTG	47.1 °C
GGTC	N.M	GCTC	N.M	GGCTC	U.W.	GCGTC	U.W.	GCCTC	U.W.

c

**5 Base pairs**			
	**Gibbs energy (kcal/mol)**	**T_m (°C)**	**T_m difference (°C)**
GGCAG	−1.42	48	+3.9
Secondary structure	−1.42	44.1	
GGCTG	−1.73	50.2	+4.6
Secondary structure	−1.73	45.6	
GCGTG	−1.24	47.1	+3.7
Secondary structure	−1.24	43.4	
GCCAG	−1.24	47.1	+3.7
Secondary structure	−1.24	43.4	
GCCTG	−1.24	47.1	+3.7
Secondary structure	−1.24	43.4	

d

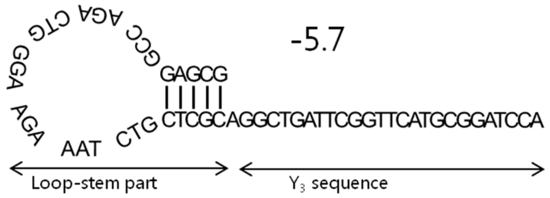

Figure S1. Theoretical evaluation of the design of the loop sequence (Y_L), a single-stranded oligo-fragment on a molecular beacon-equipped DNA nanobiosensor. (**a**) Sequences of 4–7 base pairs with a GC content of 70–80% were considered for the stem. (**b**) Loop–stem candidates that were four or five base pairs in length were analyzed by OligoAnalyzer 3.1 under the following conditions: 50 mM NaCl, 37°C, and 6 mM of each oligonucleotide. There were five possible 5-bp pair candidates. U.W. denotes unwanted hairpin-loop structure formation. The as-designed Y_L sequence is shown: its folding structures were analyzed according to thermodynamic energies using Beacon Designer. The sequence of breast-cancer-specific mRNA marker EZH2 that L-DNA targets is 5' CAGAUUUCUUCCCAGUCUGGC-3'. This sequence is complementary to the loop segment of the L-DNA, 5' GCCAGACTGGGAAGAAATCTG 3'. (**c**) Five possible candidates for combination with Y_3 were evaluated. GGCAG and GGCTG were the best candidates for linkage of the Y-DNA, because they had a low Gibbs free energy and a larger T_m difference for possible secondary structures.

A few sequence design rules should be considered when designing the loop structure of a molecular beacon. The loop–stem of the molecular beacon should be based on selection of the corresponding target sequence[1,2]. The stem should have four to seven base duplexes with a GC content of 70–80%; if RT-PCR (real-time polymerase chain reaction) is considered, the primer part should have a GC content of 50–60% (Figure S1a), and Gs and Cs should be placed at the end of primer. A guanine in the 5′ region should generally be avoided because it may quench the signal of the attached fluorophore. However, if Cy5 dye is used, a guanine can be positioned at the 5′ end with an Iowa Black®RQ quencher, but three repetitive bases of either Gs or Cs should be removed. For the loop–stem sequence, four and five bases on the stem sequence were selectively evaluated using the OligoAnalyzer 3.1 (Integrated DNA Technologies, Coralville, IA, USA)and Beacon Designer Free Edition (Premier Biosoft International, San Francisco, SF, USA). Several candidates were evaluated (Figure S1b). Five sequences that would possibly maintain a loop–stem structure around 45 °C were selected. In consideration of both Gibbs free energy and melting temperature, GCGAG and GCGTG were finally selected (Figure S1c). The loop–stem part was then linked with the Y_3 oligo-fragment. GCGAG was ultimately selected as the stem sequence, as this sequence yielded the most stable structure according to Beacon Designer software (Premier Biosoft International). The Gibbs free energy for the formation of a self-dimer was –5.8 kcal/mole, and that for a hairpin was around -5.7 kcal/mole. The values for GCGTG were similar, with a Gibbs free energy for the formation of a self-dimer of −7.2 kcal/mole and that for a hairpin of −6.0 kcal/mole. The higher Gibbs free energy of the self-dimer compared to the hairpin suggests that hairpin structure formation could be inhibited. After the loop–stem sequence was established, a Y_L sequence was proposed, as shown in Figure S1d.

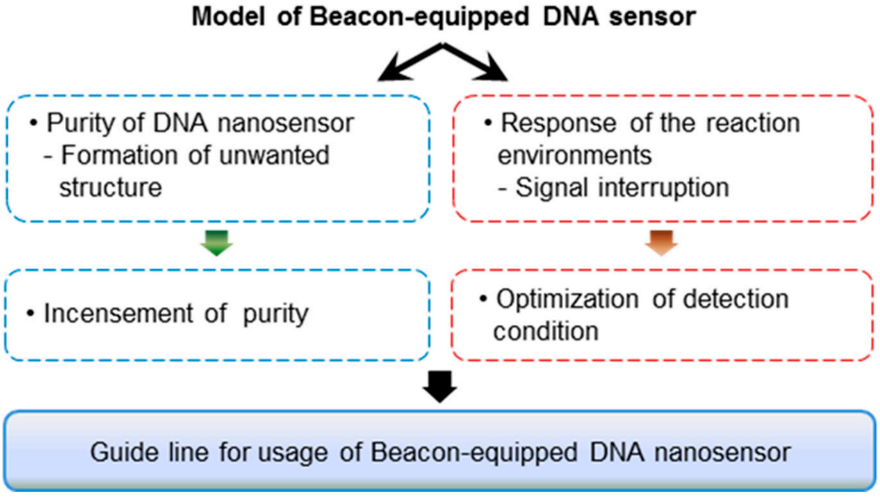

Figure S2. Algorithm diagram for optimizing the parameters for the synthesis of a beacon-equipped DNA sensor.

a

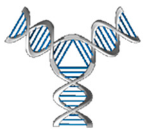

Name	Sequence
Y1	TGGATCCGCATGACATTCGCCGTAAG
Y2	CTTACGGCGAATGACCGAATCAGCCT
Y3	AGGCTGATTCGGTTCATGCGGATCCA

b

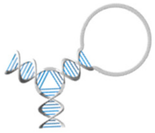

Name	Sequence
Y1	TGGATCCGCATGACATTCGCCGTAAG
Y2	CTTACGGCGAATGACCGAATCAGCCT
Y_{Loop}	GCGAGGCCAGACTGGGAAGAAATCTGCTCGC AGGCTGATTCGGTTCATGCGGATCCA

c

	**37 °C**		**50 °C**	
	**ΔG (kcal/mol)**	**T_m (°C)**	**ΔG (kcal/mol)**	**T_m (°C)**
		Y1		
1	1.29	16.60	1.86	–8.00
2	1.36	3.60	1.89	3.60
		Y2		
1	–1.57	54.20	–0.38	54.20
2	–0.32	40.10	-	-
		Y3		
1	0.50	32.80	1.68	–0.20
2	0.95	28.60	1.82	27.00

Figure S3. Sequence information for the template Y-DNA and molecular beacon-equipped DNA nanosensor, or L-DNA. A schematic and the sequence of (**a**) Y-DNA and (**b**) L-DNA. (**c**) Theoretical analysis of possible secondary structures predicted using Mfold and the RNA composer program. The secondary structures of $Y_{n\ (n=1,2\ \text{and}\ 3)}$ at 37 and 50 °C were analyzed. Gibbs free energy and T_m values of possible secondary structures are described in the table.

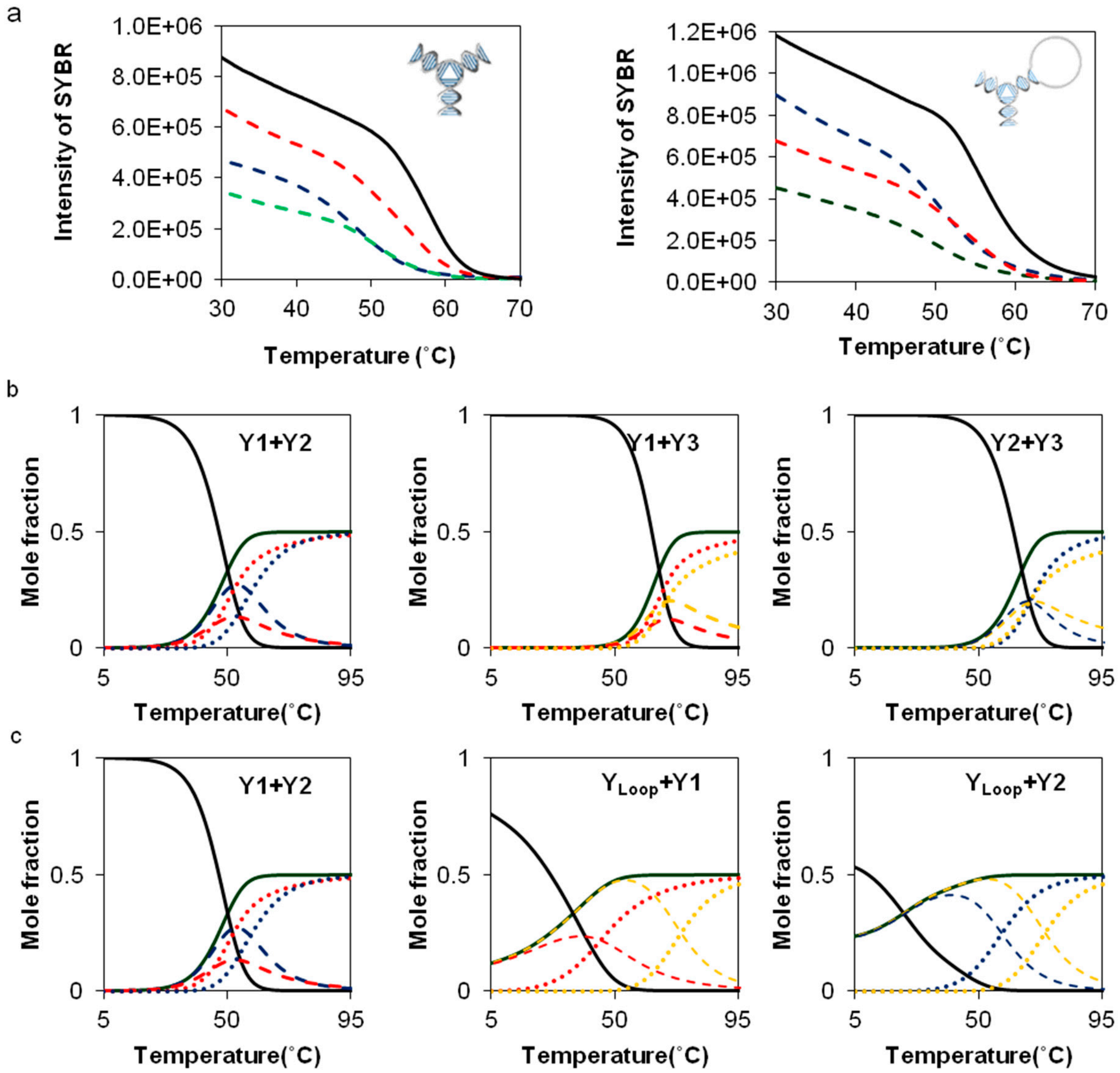

Figure S4. (**a**) Melting curve analysis of Y-DNA and L-DNA and its derivatives. Black lines indicate Y-DNA and L-DNA, whereas red, blue, and green dashed lines represent the partial and incomplete constructs of $Y_1 + Y_2$, $Y_1 + Y_3$ (or Y_L), and $Y_2 + Y_3$ (or Y_L), respectively. The T_m values of Y-DNA and L-DNA were 58 and 56.5 °C, respectively, as attained by variations in the intensity of the SYBR® green I measured by RT-PCR. Data were obtained from triplicate experiments. (**b**), (**c**) Theoretical analysis of the components of Y and L-DNA using UNAfold Mfold web server (RNA institute, Albany, NY, USA). Black and green lines indicate the combinations of two sequences and separated oligonucleotides, respectively. Dashed and dotted lines indicate the folded and unfolded states of oligonucleotides, respectively. Red, blue, and orange indicate Y_1, Y_2, and Y_3 (or Y_L), respectively. (**b**) Mole fractions of $Y_1 + Y_2$, $Y_1 + Y_3$, and $Y_2 + Y_3$ were 1.0. Whereas this data does not show the yield of Y-DNA, it suggests that theoretical yields of each combination are 100%. (**c**) Mole fractions of $Y_1 + Y_L$ and $Y_2 + Y_L$ were 0.76 and 0.53, respectively. Here, mole fraction was defined as the amount of constituent divided by total amount of all constituents in a mixture.

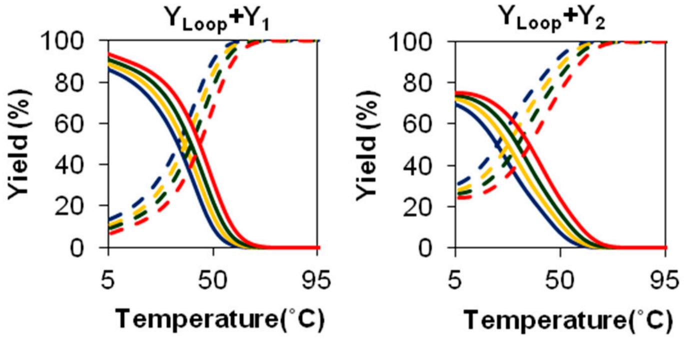

Figure S5. Yield increment in L-DNA as a function of to salt concentration. The yield of each combination was increased by different masses of salts. Solid lines indicate the binding of two oligonucleotides, and dashed lines indicate unbound oligonucleotides. Blue, orange, green, and red represent NaCl with 50, 100, 200, and 500 mM, respectively. The yield of each combination increased at high salt concentration. At a salt concentration of 500 mM, the yield of Y_{Loop} + Y1 and Y_{Loop} + Y2 increased by 7.29% and 6.10%, respectively. It is noted that the T_m (melting temperature) of a DNA sequence is a function of both enthalpy and entropy of DNA sequence and is defined as a temperature where the mass of single-stranded DNA is 50% of that of double-stranded DNA [3].

$$T_M(^\circ C) = \frac{\Delta H^\circ}{\Delta S^\circ + R\ln[\text{oligo}]} - 273.15.$$

In general, the T_m of DNA is calculated by considering nearest neighbor thermodynamic parameters [4]. However, this T_m value is changed by monovalent and divalent salt concentrations, as shown in the following equation:

$$T_M(^\circ C) = (aA + bT)x2 + (cG + dC) \times 4 - 16.6 \times \log_{10}(0.050) + 16.6 \times \log_{10}(Na^+).$$

The T_m can be revised by considering the contribution of monovalent ions using the UNAfold program:[5]

$$\frac{1}{T_M(Na^+)} = \frac{1}{T_M(1M\ Na^+)} + [(4.29 f_{GC} - 3.95)\ln[Na^+] + 0.940\ln^2[Na^+]] \times 10^{-5}.$$

For complexes of Y_L and Y_1 or Y_2, the synthesis yield as a function of salt concentration was studied using the UNAfold program.

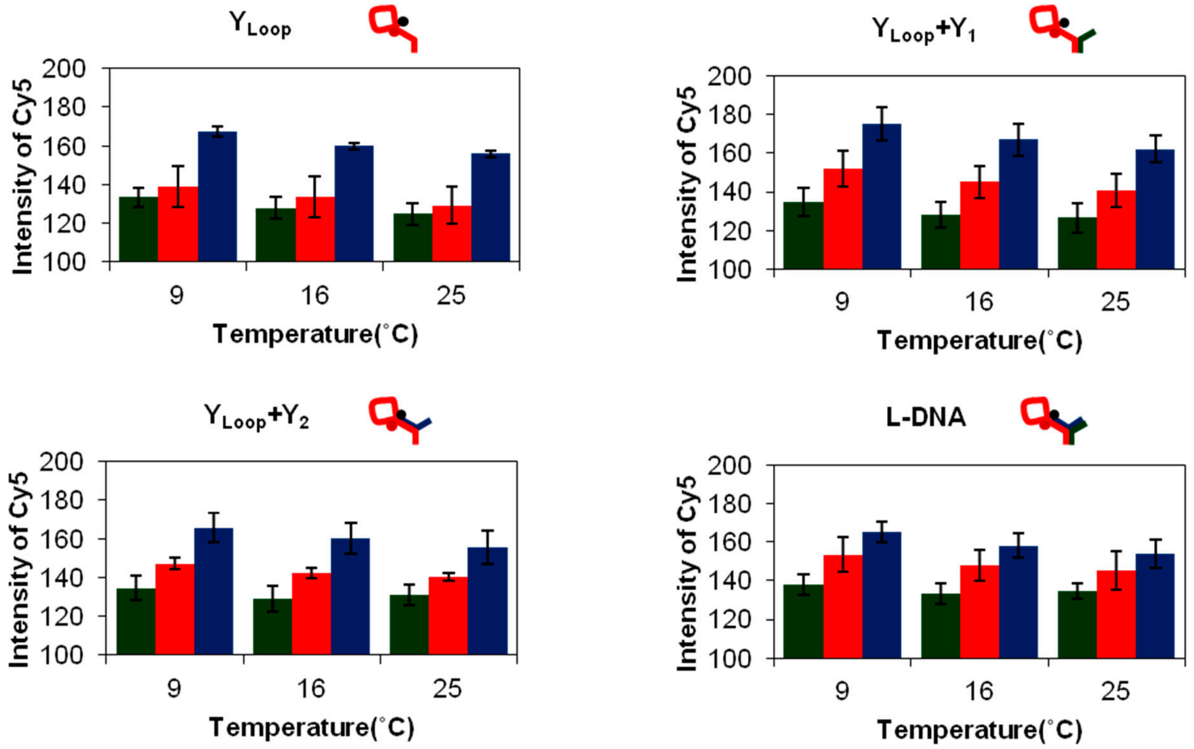

Figure S6. Detection efficiencies of L-DNA and its derivatives. Cy5 intensity of L-DNA and its derivatives at different target concentrations was evaluated at temperatures of 9, 16, and 25 °C. Green, red, and blue bars represent 0.12, 0.6, and 1.2 μM of target molecules in L-DNA solution, respectively. Intensity of Cy5 was measured spectrophotometrically. Data were obtained from triplicate experiments.

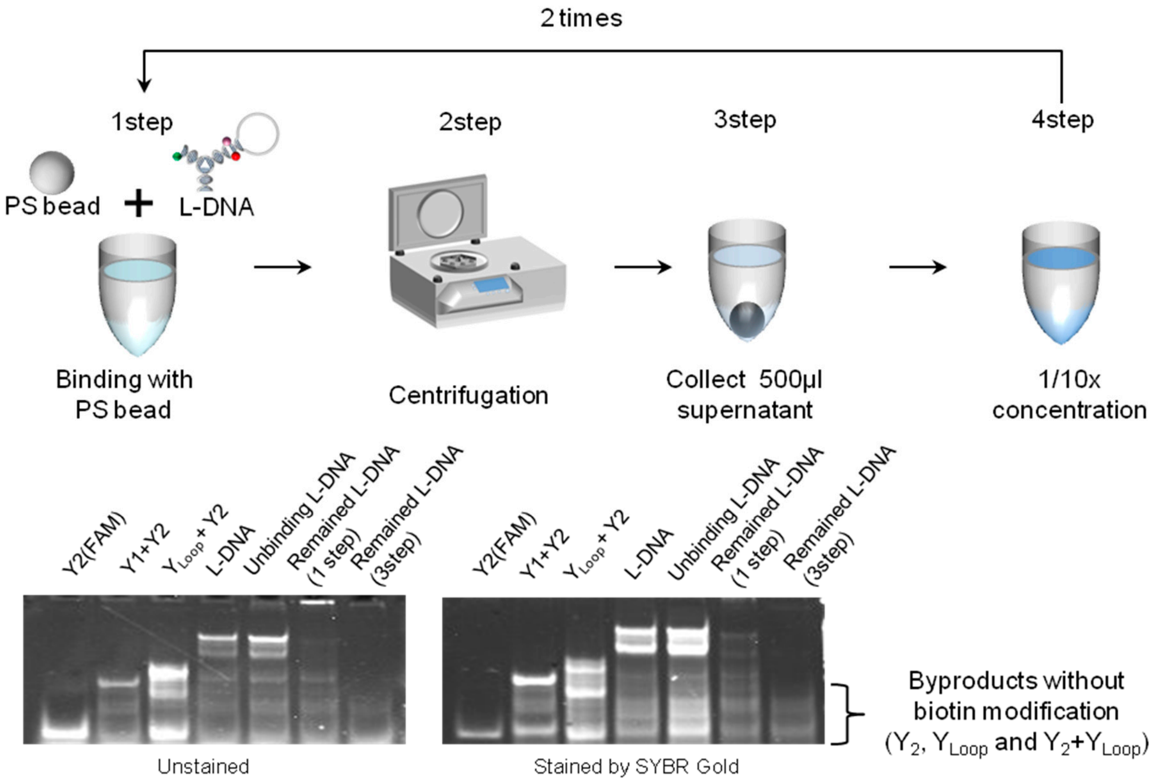

Figure S7. Synthesis of a bead-immobilized molecular beacon-equipped DNA nanobiosensor system and confirmation of unreacted sequences. To confirm unreacted sequences, the binding process was repeated three times. A 15% PAGE image of FAM-labeled L-DNA is shown. After three binding steps, the supernatant of the remaining L-DNAs consisted of Y_2, Y_L, and $Y_2 + Y_L$, but no biotin moieties.

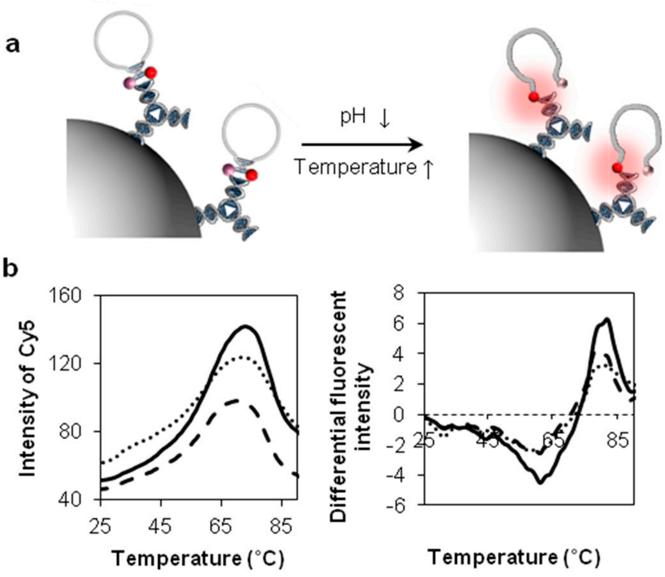

Figure S8. Environmental stability of the 110 nm sized bead-immobilized molecular beacon-equipped DNA nanobiosensor system. (**a**) Schematic of the nanobiosensor system. Stability of the looped structure changed according to pH and temperature. (**b**) Intensity of Cy5 measured at 25 °C to 90 °C in solutions of pH 5.5 (solid), 6.5 (dashed), and 7.4 (dotted). Cy5 intensity was measured by RT-PCR and expressed as the signal increment of Cy5 over that obtained at room temperature. Temperature was confirmed to affect the binding state of L-DNA and the signal intensity of Cy5. We found that a pH of 7.4 and room temperature were the optimal conditions for detecting target molecules.

Name	Sequence
miRNA 21	UAGCUUAUCAGACUGAUGUUGA
$Y_{Loop(m)}$	GCGAGTCAACATCAGTCTGATAAGCTACTCG<br>CAGGCTGATTCGGTTCATGCGGATCCA

Figure S9. Sequence information of target and Y_L for the Figure 5. $Y_{Loop(m)}$ is used instead of Y_L in L-DNA.

References

1. Monroe, W.T.; Haselton, F.R. Molecular beacon sequence design algorithm. *Biotechniques* **2003**, *34*, 68–70, 72–73.
2. Vet, J.A.; Marras, S.A. Design and Optimization of Molecular Beacon Real-Time Polymerase Chain Reaction Assays. In *Oligonucleotide Synthesis*, 1st ed.; Herdewigin, P., Ed.; Humana Press: Berlin/Leuven, Germany, 2005; pp. 273–290.
3. Allawi, H.T.; SantaLucia, J. Thermodynamics and NMR of internal G.T mismatches in DNA. *Biochemistry* **1997**, *36*, 10581–10594.
4. Roh, Y.H.; Ruiz, R.C.; Peng, S.; Lee, J.; Luo, D. Engineering DNA-based functional materials. *Chem. Soc. Rev.* **2011**, *40*, 5730–5744.
5. Owczarzy, R.; You, Y.; Moreira, B.G. Manthey, J.A.; Huang, L.; Behlke, M.A.; Walder, J.A. Effects of sodium ions on DNA duplex oligomers: Improved predictions of melting temperatures. *Biochemistry* **2004**, *43*, 3537–3554.
